# Evaluation of the merit of ethanolic extract of *Annona reticulata* as an anti-cancer agent in human colon cancer cell lines (HCT-116)

**DOI:** 10.12688/f1000research.141542.1

**Published:** 2023-12-08

**Authors:** Pooja Prakash Rao, Vijetha Shenoy Belle, Akshatha G Nayak, Nitesh Kumar, Vanishree Rao, Sri Pragnya Cheruku, Krishnananda Prabhu

**Affiliations:** 1 Department of Biochemistry, Kastubra Medical College Manipal, Maniapl Academy of Higher Education, Manipal, Karnataka, India; 2 Division of Biochemistry, Department of Basic Medical Sciences, Manipal Academy of Higher Education, Manipal, Karnataka, India; 3 Department of Pharmacology and Toxicology, National Institute of Pharmaceutical Education and Research, Hajipur, Export Promotions Industrial Park (EPIP), Industrial Area Hajipur, Vaishali, Bihar, India; 4 Department of Pharmacology, Manipal College of Pharmaceutical Sciences, Manipal Academy of Higher Education, Manipal, Karnataka, India

**Keywords:** Anti-cancer, anti-migratory, colon cancer, Annona reticulata, Phytocompounds, Gas Chromatography-Mass Spectrometry, Bioinformatics, Fractionation

## Abstract

**Background:**

Colon cancer is the fourth most common cancer type worldwide. Novel alternative therapeutic anti-cancer drugs against colon cancer with less toxicity are to be explored . This study was aimed to explore the anti-proliferative and anti-migratory activity of various fractions of *Annona reticulata* ethanolic leaf extract on human colon cancer cell lines (HCT-116) and to explore the potential molecular targets from the most potent plant extract fraction.

**Methods:**

After obtaining ethical clearance from the institutional ethics committee, the extract and fractions were prepared and a preliminary analysis of the phytochemical was done qualitatively. Total phenolic and flavonoids were determined. Ethanolic leaf extract and its fractions were subjected to cytotoxicity analysis using the sulforhodamine B assay and the most promising fraction which showed the highest viability was selected to study anti-migratory activity. The anti-migratory effect was studied using a scratch wound healing assay. Gas chromatography-mass spectrometry (GC-MS) was done to identify the major phytocompounds present in the fraction. The major five phytocompounds identified from the GC-MS were subjected to bioinformatics analysis.

**Result:**

Among the four fractions, the petroleum ether fraction exhibited the highest anti-proliferative activity. The migration of colon cancer cells was significantly inhibited by the extract and petroleum ether fraction. The major phytocompounds identified from GC-MS were phytol (13.03%), 2,6-bis (3,4-methylenedioxyphenyl)-3,7-dioxabicyclo (3.3.0) octane (11.95%), gamma.-sitosterol (10.45%), alpha.-tocopherol-beta.-D-mannoside (7.50%) and 3-amino-4-piperonyl-5-pyrazolone (5.84%). The bioinformatics analysis of these phytochemicals showed a high potential to affect the levels of key proteins driving colon cancer progression, inhibiting the enzymes and proteins overexpressed in cancer.

**Conclusion:**

The outcome of this study endorses the potential of phytochemicals of the petroleum ether fraction of ethanolic leaf extract of *Annona reticulata* for the development of a new chemotherapeutic agent in the treatment of colon cancer.

## Introduction

Colon cancer is the fourth most commonly diagnosed malignancy and the fifth leading cause of deaths due to cancer worldwide. ^1^ About 85% of the patients diagnosed with colon cancer undergo surgery with a curative intent. Even so, it is seen that the cancer recurs in about 50% of these patients even after the optimal resection of the primary tumor. ^2^ Most of the conventional chemotherapeutic treatments make use of a combination of cytotoxic drugs. However, these are usually associated with chemoresistance and side effects. ^3^ The failure of established therapies to influence the natural history of colon cancer has led the scientist to seek for new therapies based on the principles derived from cancer biology.

Herbal plants have been used extensively by the traditional healers for the treatments of several ailments, including many cancers and have been associated with fewer side effects, effective in treatment, affordable and easily available compared to the conventional chemotherapeutic drugs. ^4^ Out of 175 molecules that were approved for the treatment of cancer from the year 1940 to 2017, 49% were natural or modified natural products, which indicates the significant contribution of the natural phytochemicals towards the development of the anticancer agents. ^5^

Previous studies have shown that alcoholic leaf extract of *Annona reticulata* exhibits anticancer activity against various cancers such as hepatoma, melanoma, colon cancer, cervical cancer, lung cancer and breast cancer. ^6^ Previous studies using ethanolic leaf extract of *Annona reticulata* have significantly inhibited 1,2-dimethylhydrazine (DMH) induced colon cancer in rats. ^7^

The present study was carried out to identify the phytocomponents and to explore the anticancer activity of ethanolic leaf extract *Annona reticulata* and its fractions on its anti-proliferative and anti-migratory activities on HCT-116 colon cancer (adenocarcinoma) cell lines.

## Methods

### Ethical clearance and study site

The study was carried out after getting approval from the Institutional ethics committee (IEC - 723/2019) between 1 ^st^ November 2019 to 25 ^th^ May 2021.

### HCT-116 colon cancer (adenocarcinoma) cell lines

HCT-116 colon cancer cell lines were purchased from NCCS (National Centre for Cell Science), Pune, India and cultured on Dulbecco’s Modified Eagle Medium (procured from Invitrogen, Thermo Fisher Scientific, India) containing penicillin (100 units/mL), streptomycin (100 μg/mL) and 10% fetal bovine serum (FBS) in a humidified incubator (Rivotech Incubator, India) maintained at 37°C and 5% CO _2_ for 24-48 hours. The plates can be stored for 6 months at -80°C for future use.

### Plant material collection and authentication

The leaves of *Annona reticulata* were procured from the herbal garden of Dhanya Nursary, Koteshwara, Karnataka, India. The authentication of the plant was performed by the Department of Plant Sciences, Bangalore Central University, Karnataka, India.

### In-vitro methodology

*Preparation of ethanolic leaf extract of Annona reticulata*

Fresh leaves of *Annona reticulata* (1 kg) were cleaned using water and air dried for 20 days. Then the leaves were homogenized and subjected to grinding using a blender (Panasonic MX-AC400 1000-Watt Mixer Grinder) to obtain a fine powder. This leaf powder was macerated in a Soxhlet apparatus (Borosil, India) with ethanol (1L) until the solvent became colorless. After the extraction was complete, using a rotary vacuum evaporator (EPS Biosolutions, India), the solvent was evaporated until semisolid crude extract was obtained. This ethanolic leaf extract was stored at -20°C until use. ^8^

*Fractionation of ethanolic leaf extract of Annona reticulata*

The ethanolic leaf extract (100 g) was sequentially fractionated using a series of organic solvents starting with petroleum ether followed by diethyl ether, chloroform, and ethyl acetate in a fractionating column (Borosil, India). The extract obtained with each solvent was filtered using a rotary vacuum evaporator (EPS Biosolutions, India) at 250-280 rpm. The evaporation of the solvent was carried out at a temperature below 35°C. ^9^

*Preliminary phytochemical analysis of ethanolic leaf extract and its fractions*

*Annona reticulata* ethanolic leaf extract and its fractions were evaluated for the presence of various classes of phytocomponents using specific biochemical tests mentioned below. ^10^^,^^11^

*Test for Terpenoids*

2 mL of Chloroform was added with the 5 mL of alcohol leaf extract. Then 2 mL of concentrated sulphuric acid was added to the solution. If a reddish-brown color formed this would indicate the presence of terpenoids.

*Test for Flavonoids*

To test for flavonoids, 2 mL of 20% NaOH mixture was mixed with 2 mL of alcohol leaf extract for the development of the yellow color. If the solution turns colorless after the addition of 2 drops of diluted acid confirms the presence of flavonoid in the given sample.

*Test for Phenols/polyphenols*

2 mL of test solution in alcohol was added with one drop of neutral ferric chloride 5% solution. The formation of an intense blue color indicates the presence of phenols.

*Test for Saponins/fatty acids*

5 mL of distilled water was mixed with 1 mL alcohol leaf extract in test tube and it was mixed vigorously using vortex mixer. The upper froth layer was separated and mixed with few drops of olive oil and mixed vigorously and the foam appearance showed the presence of saponins.

*Test for Tannins*

2 mL of test solution was added with bromine water and acetate. Decoloration of bromine water indicates the presence of tannins.

*Test for steroids/polysterols*

2 mL of chloroform and concentrated sulphuric acid were added with the 5 mL leaf extract. In the lower chloroform layer red color would appear to indicated the presence of steroids.

*Test for Alkaloids - Drangendroff’s test*

2 mL of the extract was mixed with 1 mL of Dragendroff’s reagent. The formation of orange or orange red precipitate indicates the presence of alkaloids.

*Test for carbohydrates*

The Anthrone test was used in this study. 2 mg of ethanolic extract was shaken with 10 mL of water, and the filtrate was concentrated. To this 2 mL of anthrone reagent solution was added. Formation of green or blue color indicates the presence of carbohydrates.

*Analysis of total phenolic and flavonoid content of ethanolic leaf extract and its fractions*

Total phenolic content (TPC) and total flavonoid content (TFC) of ethanolic leaf extract and its fractions were determined using the Folin-Ciocalteu method and aluminum chloride colorimetric assay respectively. ^12^ In brief, to analyze TPC, 12.5 μl of *Annona reticulata* ethanolic leaf extract and its fractions (1 mg/mL) was added to a 96-well microplate [Thermo Fisher Scientific, India] and mixed with 125 μL of Folin–Ciocalteu reagent and the mixture was incubated for 5 minutes at room temperature. This was followed by the addition of 12.5 μL of 7% Na _2_CO _3_ and the plate was placed in the dark for 90 min. With the help of a microplate reader (Thermo Fisher Scientific, India) the absorbance was measured at 760 nm. Further, utilizing the standard curve of gallic acid, TPC was determined, and the results were expressed as mg of gallic acid equivalent per gram (mg GAE/g) of dry plant material.

For the analysis of TFC, briefly, 100 μL of *Annona reticulata* ethanolic leaf extract and its fractions (1 mg/mL) was added to a 96-well microplate (Thermo Fisher Scientific, India) and mixed with 100 μL of 2% aluminum chloride, and the final mixture was incubated for 10 min. The absorbance was measured using a microplate reader (Thermo Fisher Scientific, India) at 368 nm. Drawing upon the curve of quercetin, the TFC was determined, and the result was expressed as mg quercetin equivalent per gram (mg QE/g) of dry plant material.

*Gas chromatography-mass spectrometry (GC-MS) analysis of petroleum ether fraction of ethanolic leaf extract of Annona reticulata* ^13^^,^^14^

The GC-MS analysis of the petroleum ether fraction was performed using a gas chromatography-mass spectrometer equipped with an RTX5 capillary column (length: 30 m×0.25 mm; film thickness: 0.25 micron) (RESTEK, PA, USA). An aliquot of 1 μl of the petroleum ether fraction of *Annona reticulata* ethanolic leaf extract was injected into the capillary column. Helium gas was used as a carrier gas at a flow rate of 1.0 ml/min. Initially, the temperature of the column was set at 60°C for 2 minutes and then was raised to 150°C and maintained at that temperature for 5 minutes. Then the temperature of the oven was increased and maintained at 280°C for 10 min. The ion source temperature was fixed at 200°C and the interface temperature was set at 280°C. Mass spectral scan range of 40-500 m/z was attained. The relative percent quantity of each phytocomponent in the petroleum ether fraction was evaluated by comparing the average peak area of each component to the total area of the chromatogram. The components present in the extract based on the GC-MS result were identified using Wiley-8 library and National Institute of Standard and Technology Mass Spectral Library (NIST). This analysis was carried out at Analytical Research & Metallurgical Laboratories Pvt. Ltd. (ARML), Bengaluru, India.

*Growth conditions and Subculture of HCT116 colon carcinoma cells*

HCT116 is an adherent human colorectal carcinoma cell line and has an epithelial morphology. The cells were cultured in T-25 (Eppendorf, India) flasks in DMEM, high glucose consisting of 10% Fetal Bovine Serum [FBS]. The cells are grown until 60 to 70% confluency is achieved upon which, the cells are trypsinized by addition of 1ml of 0.2% Trypsin-EDTA solution ((Gibco ^TM^, ThermoFischer Scientific, India). The detached cells were collected and counted using conventional neubaur chamber for seeding. The seeding density was dependent on the surface area of the well.

*Sulforhodamine B assay*

Sulforhodamine B (SRB) assay was carried out to determine the effect of the ethanolic leaf extract and its fractions on the viability of the HCT-116 cell line. ^15^ HCT116 cells were seeded in a 96-well plate at a density of 5000 cells/well. The cells were given a 24h window to adhere to the well surface, after which, the cells were treated with compounds of interest at different concentration ranges. The ethanolic leaf extract and its fractions were added at a concentration range of 500 μg/mL - 7.81 μg/mL for a period of 48h to evaluate cytotoxic effect. 0.1% DMSO was used as control as the stock was prepared in DMSO such that, the highest concentration added to the well contains ≤0.1% DMSO. The standard anticancer drug, doxorubicin was added to the wells at concentration range of 24 μM - 0.046 μMand was used as positive control in the assay. Measurement of absorbance was carried out at 510 nm with the help of a microplate reader (Thermo Fisher Scientific, India), and the dose-response curve was used to calculate the half maximal inhibitory concentration (IC _50_) of each fraction. The most promising fraction was then chosen for further studies.

*Scratch wound healing assay for migration of HTC-116 cell lines*

Scratch wound assay was performed to evaluated the migratory properties of cancer cells in response to treatment with our prepared extracts. A monolayer of cells were prepared by seeding 2×10 ^5^ HCT-116 cells/mL in a 6-well plate and allowed to adhere to the well surface during a 24 hr window. Following this, Dulbecco’s phosphate buffered saline (DPBS) was used to wash the cells and then using a sterile 200 μl micropipette tip, a linear wound was created in the cell monolayer. DPBS was used to remove any detached cells and cellular debris. ^16^ *Annona reticulata* ethanolic leaf extract (30.16 μg/mL) and petroleum ether fraction (29.07 μg/mL) prepared by appropriate dilution with serum-free DMEM was added to the wells in duplicates To evaluate their ability to inhibit the migration of the cells. The concentrations of the ethanolic leaf extract and petroleum ether fraction used were based on the IC _50_ values of these fractions as obtained from the sulforhodamine B cytotoxicity assay. Wound areas were photographed at 0 and 24 hr in 4× magnification using an Labomed (USA, Model -TCM 400) inverted tissue culture microscope with a digital camera. The cells treated with 0.1% DMSO served as control and cells treated with doxorubicin (0.75 μM) served as a positive control. The images were analyzed and photographed at two time points that is at ‘0’ hr (zero) and after 24 hr of treatment using ImageJ software (NIH, USA) RRID:SCR_003070 version 1.53j. The healing of the wound was considered as closure of the wound and rate of migration of the cells was represented as percentage (%) migration for each group and compared with DMSO control

$$\text{\% Migration rate}=\left(\frac{A_{i}-A_{24\;\mathrm{hr}}}{A_{i}}\right)\times 100$$

Where, *A*_*i*_ is the initial wound area at ‘0’ (zero) hr and *A*_24hr_ is the wound area after 24 hr of extract/drug administration.

### Bioinformatics analysis

Bioinformatics analysis was carried out to determine the potential molecular targets of the major phytocompounds of the most potent fraction. The structures of the top five phytocompounds from GC-MS data of the petroleum ether fraction were retrieved from the NCBI PubChem database. These structures were then uploaded to the prediction of activity spectra for substances (PASS) online server (Version 2.0) to analyze the potential anticancer activities of active compounds using target fishing screening. This screening was based on the chemical similarity between various molecules and on the utilization of current knowledge of the biological activity of small molecules. “Chemical similarity principle” stating that similar molecules are likely to possess equivalent properties form the basis of this methodology. The potential anticancer activity of a phytocomponent using the PASS online server was determined on the basis of the probability activity (Pa) values of the pharmacological properties in the range of 0.03 to 0.99. The results were then filtered for anticancer activity, analyzed and recorded. ^17^

### Statistical analysis

All the assays were carried out in triplicate (n=3). The data was analyzed using graph pad prism (V.8.4.3) software with a perpetual license and the data from all the assays are expressed as mean ± standard deviation. One way-Analysis of Variance (ANOVA) followed by Duncan’s multiple range post hoc test was used to determine the statistical significance within various fractions. Student t-test was used to analyze the statistical significance between the treatment and control groups and Duncan’s multiple range test was used to analyze the statistical significance within various groups. *p*<0.05 was considered statistically significant.

## Results

### Fractionation of ethanolic leaf extract of Annona reticulata

The final dried ethanolic leaf extract yield was 170.3 g and was stored at -20°C until use. Fractionation of ethanolic leaf extract produced dried petroleum ether fraction (0.5 g), diethyl ether fraction (15.06 g), chloroform fraction (47.07 g) and ethyl acetate fraction (35.1 g).

### Preliminary phytochemical analysis

Results of the phytochemical analysis of ethanolic leaf extract of *Annona reticulata* and its fractions are summarized in Table 1. The presence of terpenoids, flavonoids, polyphenols, tannins, steroids and poly-sterols were reported in the extract, whereas alkaloids and saponins were absent.

**Table 1.** Phytochemical analysis of *Annona reticulata* ethanolic leaf extract and its fractions.

Phytochemical constituents	Ethanolic leaf extract	Petroleum ether fraction	Diethyl ether fraction	Chloroform fraction	Ethyl acetate fraction
**Alkaloids**	-	-	-	-	-
**Terpenoids**	+	+	-	-	-
**Flavonoids**	+	+	+	+	+
**Carbohydrates**	+	-	-	-	-
**Polyphenols**	+	+	+	+	+
**Tannins**	+	+	+	+	+
**Proteins**	-	-	-	-	-
**Saponins**	-	-	+	+	+
**Polysterols**	+	+	-	-	-
**Steroids**	+	+	-	-	-
**Fatty acids**	+	+	+	-	-

Legends: “+” indicates presence; “-” indicates absence.

### Total phenolic and flavonoid content of ethanolic leaf extract of *Annona reticulata* and its fractions

TPC was expressed as mg of gallic acid equivalent per gram of dry plant material (mg GAE/g) and TFC was expressed as mg of quercetin equivalent per gram of dry plant material (mg QE/g). Ethanolic leaf extract was found to contain highest TPC (46 ± 0.26 mg GAE/g of plant extract) and TFC (19.9 ± 0.3 mg QE/g of plant extract) compared to its fractions. The TPC and TFC of all the fractions were mentioned in Table 2.

**Table 2.** Total phenolic and flavonoid content of *Annona reticulata* ethanolic leaf extract and its fractions.

Extract/fraction	Ethanolic leaf extract (Mean ± SD)	Petroleum ether fraction (Mean ± SD)	Diethyl ether fraction (Mean ± SD)	Chloroform fraction (Mean ± SD)	Ethyl acetate fraction (Mean ± SD)
**TPC (mg GAE/g of plant extract)**	46 ± 0.26	13.5 ± 0.19	18.5 ± 0.28	23.5 ± 0.12	29.07 ± 0.28
**TFC (mg QE/g of plant extract)**	19.9 ± 0.3	2.02 ± 0.26	4.2 ± 0.17	8.97 ± 0.12	14.21 ± 0.05

Legends: The results are represented as mean ± SD (standard deviation) for triplicate samples (n=3). TPC: Total phenolic content; TFC: Total flavonoid content; mg GAE/g: milligrams of gallic acid equivalents per gram of plant extract; mg QE/g: milligrams of quercetin equivalents per gram of plant extract.

### GC-MS analysis of the petroleum ether fraction

**Figure 1.**
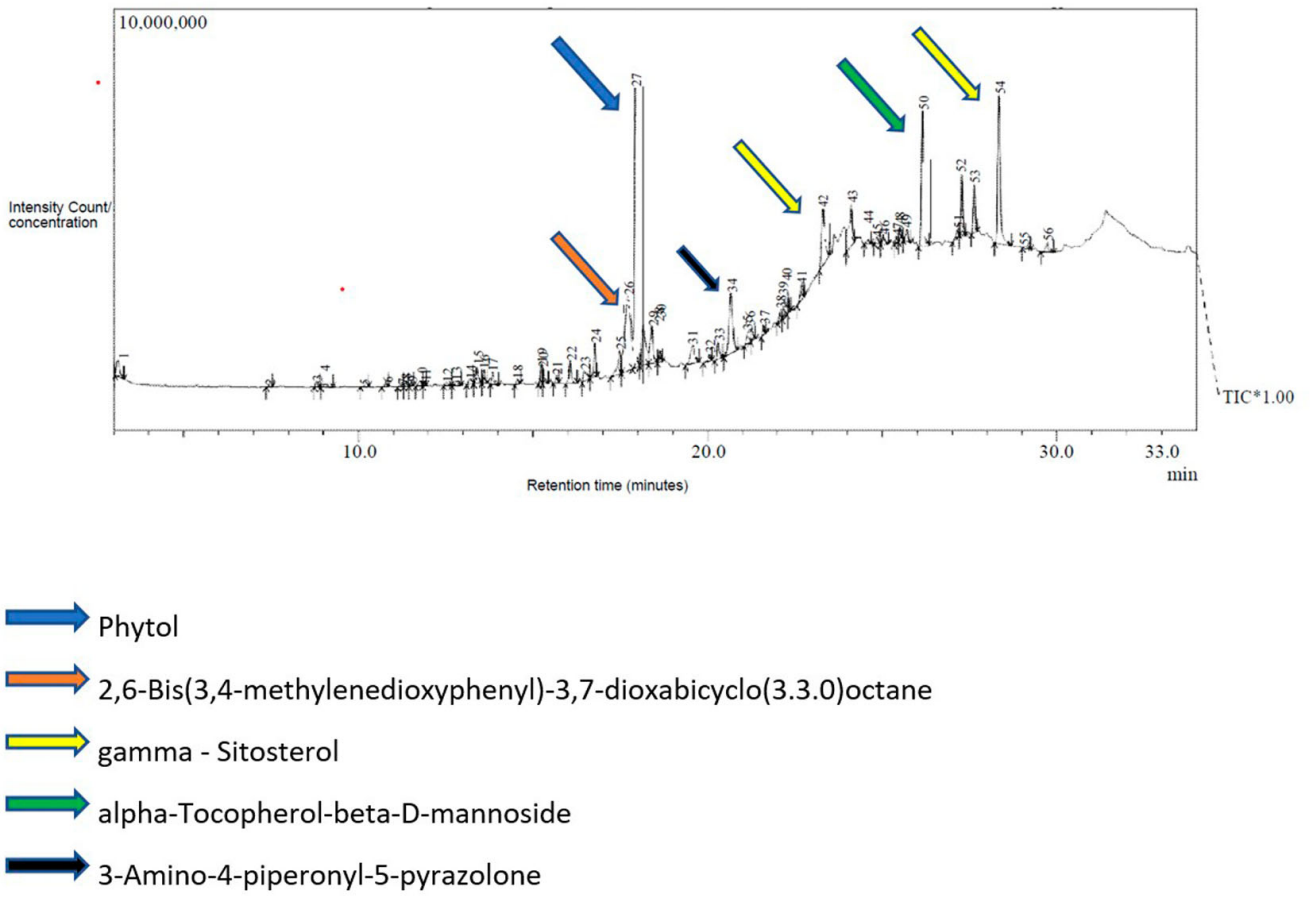
Chromatogram of GC-MS analysis showing various phytoconstituents in petroleum ether fraction of ethanolic leaf extract of *Annona reticulata.*

The GC-MS analysis of the petroleum ether fraction revealed the presence of 56 volatile compounds ( Figure 1). The most predominant phytocompounds present in the petroleum ether fraction were phytol (13.03%), 2,6-bis (3,4-methylenedioxyphenyl)-3,7-dioxabicyclo (3.3.0) octane (11.95%), gamma.-sitosterol (10.45%), alpha.-tocopherol-beta.-D-mannoside (7.50%) and 3-amino-4-piperonyl-5-pyrazolone (5.84%) were labeled and indicated in Figure 1.

### Effect of ethanolic leaf extract and its fractions on proliferation of HCT-116 colon cancer cells

The dose-dependent curve equations were used to determine the IC _50_ for ethanolic leaf extract and its fractions. *Annona reticulata* ethanolic leaf extract and its fractions significantly ( *p*<0.05) inhibited the proliferation of HCT-116 cells in a dose-dependent manner ( Table 3). The ethanolic leaf extract demonstrated a potent anti-proliferative activity with an IC _50_ of 30.16 ± 0.56 μg/mL. Among the fractions tested, petroleum ether fraction exhibited the highest anti-proliferative activity (IC _50_-29.07 ± 0.83 μg/mL) followed by diethyl ether fraction (IC _50_-32.03 ± 0.78 μg/mL), ethyl acetate fraction (IC _50_-33.66 ± 1.20 μg/mL) and chloroform fraction (IC _50_-43.0 ± 1.64 μg/mL).

**Table 3.** Effect of ethanolic leaf extract of *Annona reticulata* and its fractions on cytotoxic assay for cell viability on HCT-116 cells.

Treatment	Concentration range	Inhibitory concentration _50_ (IC _50_) values ^$^
Ethanolic leaf extract	500-7.81 (μg/mL)	30.16 ± 0.56 (μg/mL)
Petroleum ether fraction	500-7.81 (μg/mL)	29.07 ± 0.83 (μg/mL)
Diethyl ether fraction	500-7.81 (μg/mL)	32.03 ± 0.78 (μg/mL)
Chloroform fraction	500-7.81 (μg/mL)	43.04 ± 1.64 (μg/mL)
Ethyl acetate fraction	500-7.81 (μg/mL)	33.66 ± 1.20 (μg/mL)
Doxorubicin	24-0.046 μM	0.75 ± 1.09 μM

Legends: The results are represented as mean ± SD (standard deviation) for triplicate samples (n=3) where

^$^ represent IC _50_ values at which maximum anti-proliferative effect was observed.

Based on the results of the anti-proliferative assay, ethanolic leaf extract and petroleum ether fraction were chosen for further studies.

### Effect of ethanolic leaf extract and petroleum ether fraction on the migration of HCT-116 colon cancer cells

**Figure 2.**
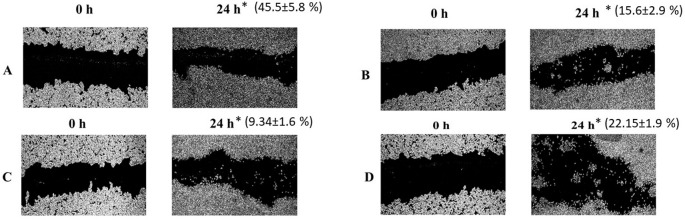
Effects of *Annona reticulata* ethanolic leaf extract, petroleum ether fraction and doxorubicin on cancer cell migration. Legends: The images demonstrate the inhibition of cell migration of HCT-116 cells where A represents DMSO control (0.1%), B represents ethanolic leaf extract at its IC _50_ (30.16 μg/mL), C represents petroleum ether fraction at its IC _50_ (29.07 μg/mL) and D represents Doxorubicin at its IC _50_ (0.75 μM). The images are captured under 40x magnification at ‘0’ hour and 24 hours of treatment with ethanolic leaf extract, petroleum ether fraction, standard drug (doxorubicin), where * represent percentage of inhibition of migration after 24 hours of treatment.

There was significant reduction in the migration of HCT-116 colon cancer cells into the wound area upon the treatment with *Annona reticulata* ethanolic leaf extract ( *p*<0.05, Figure 2B) and its petroleum ether fraction ( *p*<0.01, Figure 2C) when compared to standard drug doxorubicin ( Figure 2D) at the end of 24 h.

The migration rate of HCT-116 cells was 15.6 ± 2.9% and 9.34 ± 1.6% at 24 h post treatment with ethanolic leaf extract (30.16 μg/mL) and petroleum ether fraction (29.07 μg/mL) respectively. The petroleum ether fraction demonstrated highest anti-migratory activity on HCT-116 cells compared to ethanolic leaf extract and standard drug doxorubicin ( Figure 2).

### Bioinformatics analysis of major phytocomponents of the petroleum ether fraction

The bioinformatics analysis of the five major phytocompounds of the petroleum ether fraction using the PASS online server tool revealed the potential pharmacological activities and molecular targets, indicating the possible interactions of these compounds in cancer signaling pathways. A higher Pa value indicates greater probability of the pharmacological activity. Based on the chemical similarity principle it was seen that the phytocompounds of the petroleum ether fraction have the ability to inhibit enzymes and proteins overexpressed in cancers such as EIF4E, B-Raf, NOS2, ICAM, caspase 3, caspase 8, and MMP-9, demonstrating their potential to inhibit various processes in cancer such as proliferation, migration, invasion, angiogenesis and immune invasion. The phytochemicals also showed a high potential to affect the levels of key proteins driving colon cancer progression such as c-Myc, MMP-9, p53 and B-Raf indicating their potential to be developed as promising anticancer agents for colon cancer. The predicted anticancer activity and the possible molecular targets of these compounds are given in supplementary file.

## Discussion

One of the major causes of colon cancer mortality is metastasis and it involves multiple sequential and integrated cellular processes. ^18^ Given the importance of cell migration and angiogenesis in tumor metastasis, the development of chemotherapeutic agents targeting the pathways and proteins involved in these processes can prove to be effective for the treatment of metastatic cancers. ^19^ Natural phytocompounds are an important source of emerging chemo-preventive, chemotherapeutic and chemo-sensitizing agents for the treatment of cancer. ^20^

Plant polyphenols and flavonoids were reported to have numerous biological effects including anti-inflammatory, antioxidant, antiproliferative, and anticarcinogenic properties and have shown to possess the potential to be developed into relatively safe and effective chemotherapeutic agents. ^21^ It was seen that the amount of total phenolic and flavonoid content of the fractions increased with the increase in polarity of the solvent reported in this study. These findings were supported by previous studies which have shown that the solubility of polyphenolic compounds is higher in organic solvents with polarity lower than that of water. ^22^^,^^23^ Further, the polar properties of the polyphenols determine their solubility and the relative lipophilicity. ^24^ The total phenolic content of *Annona reticulata* ethanolic leaf extract and its fractions was higher than that of previously reported for other members of the Annonaceae family such as ethanolic leaf extracts of *Annona muracata* and *Monodora tenuifolia.* ^25^

One of the most important properties of the chemotherapeutic agents is the ability to inhibit the proliferation of cancer cells and induce apoptosis. ^26^ All the fractions of *Annona reticulata* ethanolic leaf extract exhibited anti-proliferative activity towards HCT-116 cell line in a dose-dependent manner. In the *in-vitro* SRB assay, the least polar solvent fraction, *i.e.,* petroleum ether fraction showed the highest anti-proliferative effect towards HCT-116 cells as evidenced by its lowest IC _50_ value amongst other solvent fractions, despite of containing the lowest TPC and TFC among others. This indicates that relatively non-polar phytocompounds present in the petroleum ether fraction such as alpha-tocopherol-beta-D-mannoside and gamma-sitosterol contribute significantly to the anticancer activity. As per the American National Cancer Institute, a crude extract’s IC _50_ value of less than 30g/mL is regarded promising, ^27^ signifying the potential of the petroleum ether fraction and its phytocompounds for the treatment of colon cancer. At IC _50_ concentration, the cytotoxicity induced by *Annona reticulata* ethanolic leaf extract and its petroleum ether fraction on HCT-116 colon cancer cell line was significantly higher than that induced by its ethanolic root extract on A-549, K-562, HeLa, and MDA-MB cell lines. ^28^ Based on previous studies it was observed that HCT-116 colon cancer exhibited higher sensitivity for ethanolic leaf extract of *Annona reticulata* than that for ethanolic extract of *Zingiber officinale*, essential oils of *Illicium verum* and methanolic branch extract of *Anacardium occidentale.* ^29^ The high anti-proliferative property exhibited by the petroleum ether fraction of *Annona reticulata* ethanolic leaf extract towards the colon cancer cells could be attributed to the presence of a diverse class of phytochemicals in it, as demonstrated by the GC-MS analysis carried out in this study. Based on the chemical similarity principle, phytocompounds like phytol, 2,6-bis(3,4-methylenedioxyphenyl)-3,7-dioxabicyclo (3.3.0) octane, and gamma-sitosterol have the potential to function as JAK2 expression inhibitor and B-Raf inhibitor thereby inhibiting STAT activity through the JAK/STAT pathway and inhibit the signaling through the RAS/RAF/MEK/ERK pathway respectively, reducing the expression of genes intricated in cancer cell proliferation thereby inducing the anti-proliferative effect. ^30^^,^^31^ The ability of these phytocomponents to act as potent antioxidants also contributes to the inhibition of proliferation of cancer cells. ^32^

The process of cell migration and invasion through the extracellular matrix (ECM) is an important step in cancer metastasis and is a hallmark of cancer. ^33^ For metastasis, cancer cells have to migrate and invade the basement membrane, ECM and the endothelial cell layer in order to reach the blood or lymphatic vessels. ^34^ Hence, complementation of colon cancer treatment with drugs that can inhibit the ability of the cancer cells to migrate and invade the ECM would aid in inhibiting the establishment of secondary tumors through metastasis. ^35^ In this study, *Annona reticulata* ethanolic leaf extract and its petroleum ether fraction significantly inhibited the migration of the HCT-116 cells, which was observed as a decrease in the rate of cell migration. The anti-migratory activity exhibited by the extract and its fraction on colon adenocarcinoma cell lines was found to be higher than that observed by previous studies for the compounds thymol, kaempferol and wogonin. ^36^ The phytoconstituents in petroleum ether fraction, 2,6-bis (3,4 methylenedioxyphenyl)-3,7- dioxabicyclo (3.3.0) octane can modulate the expression of cell cycle regulators and suppress the expression of eukaryotic translational initiation factor 4E (EIF4E). This mechanism is responsible for inhibition of expression of an ECM degrading enzyme, matrix metalloprotease-9 (MMP-9), thereby preventing cancer cell metastasis. ^35^ Besides this, inhibition of nitric oxide synthase-2 (NOS2) expression by the phytochemicals of the petroleum ether fraction could also be one of the possible mechanisms involved in its anticancer property by preventing cancer cell migration. ^37^

GC–MS analysis was performed for the petroleum ether fraction showed the highest anti-proliferative and anti-migratory activity amongst the other fractions tested. The most abundant phytochemical of the petroleum ether fraction was phytol, an acyclic diterpene alcohol and has proven to possess cytotoxic activity against several cancers. ^38^^,^^39^ The other two phytocompounds found in high quantities were 2,6-bis(3,4-methylenedioxyphenyl)-3,7-dioxabicyclo(3.3.0) octane and gamma.-sitosterol. Studies have proven that these compounds inhibit tumorigenesis, tumor cell proliferation, growth and survival, thereby inhibiting various cancers. ^40^ 3-Amino-4-piperonyl-5-pyrazolone is a compound that contains a piperonyl ring and previous studies have shown that the compounds containing piperonyl ring were known to possess anti-cancer properties. ^41^ Proteins like p53 and NF-κB are involved in colon cancer progression ^42^ and docking studies have shown that alpha.-tocopherol-.beta.-D-mannoside interact with these compounds and inhibit their action, thereby prevent cancer cell progression. ^43^ Hence the potent anticancer activity exhibited by the petroleum ether fraction can be attributed to the presence of these phytocompounds independently or to their synergistic effects.

The plant phytocompounds derived tumor-targeting complexes provide hope for synthesizing natural anti-cancer agents that are more effective and that exhibit specific toxicity towards tumors but remain relatively non-toxic to healthy tissues in the body. ^44^ Once the proteins involved in tumor growth, proliferation and metastasis of colon cancer were identified using the NCBI PubMed database, based on the structure of the major compounds found from GC-MS analysis and the chemical similarity principle, we have identified that the phytocompounds from the petroleum ether fraction of *Annona reticulata* ethanolic leaf extract can potentially target specific genes, regulatory proteins and enzymes involved in colon cancer progression such as c-MYC, TP53, JAK2, caspase 3, caspase 8, NOS2 *etc.* These results indicate the ability of phytocompounds in the petroleum ether fraction of *Annona reticulata* ethanolic leaf extract to inhibit various steps of colon cancer progression and metastasis such as cell migration, invasion and angiogenesis, thus signifying their potential to be developed into novel anticancer agents. These targets can further be studied using *in-vitro* and *in-vivo* studies for the development of novel targeted therapies.

## Conclusions

*Annona reticulata* ethanolic leaf extract and its fractions exhibited potent anticancer activity against colon cancer as evidenced by their significant anti-proliferative and anti-migratory HCT-116 colon cancer cells. Based on the observations from the *in-vitro* studies it appears that the phytocompounds in the petroleum ether fraction of *Annona reticulata* ethanolic leaf extract may have potential anti-cancer properties which should be further explored and experimented for its possibility to be developed as a novel anti-cancer/neo adjuvant chemotherapeutic agent. Further studies can be carried out to study the mechanism of cell death.

### Ethical considerations

The study was carried out after getting approval from the Institutional ethics committee (IEC - 723/2019).

## Data Availability

Figshare: Data files, https://doi.org/10.6084/m9.figshare.24124425.v1. ^45^ This project contains the following underlying data:
- Plant sample-GC MS data.pdf - 9,12 octadecadienoic acid.docx - 9,12octadecadienoic acid,dihydroxypropyl ester.docx - Ethyl alpha d glucopyranoside.docx - Hexadecanoic acid.docx - linoleic acid ethyl ester.docx - SRB data of extract.xlsx Data are available under the terms of the Creative Commons Attribution 4.0 International license (CC-BY 4.0).
