# Supplementary material for: Evaluation of the merit of ethanolic extract of Annona reticulata as an anti-cancer agent in human colon cancer cell lines (HCT-116)
Source: F1000Res. 2024 Jun 7;12:1571. Originally published 2023 Dec 8. [Version 2] doi: 10.12688/f1000research.141542.2 (PMC11759889; doi:10.12688/f1000research.141542.2)
Supplement: Peer review history

# Reviewer report: Reviewer response for version 2

**DOI:** 10.5256/f1000research.167401.r341405

**Published:** 2025-01-24

**Authors:** Mohd Adzim Khalili Rohin

**Affiliations:** 1 School of Nutrition and Dietetic, Faculty of Health Sciences, Universiti Sultan Zainal Abidin (UniSZA), Kuala Nerus, Malaysia

The manuscript has been accepted. 

 Manuscript version 2 was submitted after the modifications suggested by Reviewer 1. Modifications included a few significant comments and minor comments.

 In the **‘Abstract,’** the global incidence of colon cancer was updated as per the latest reference.

**‘Introduction’** was modified with the latest references and provided details on ‘the traditional use of *Annona reticulata*’.

**‘Materials and methods’** were modified by giving details on plant sample collection.

 The latest ‘ **References**’ were updated wherever applicable in the manuscript.

Is the work clearly and accurately presented and does it cite the current literature?

Yes

If applicable, is the statistical analysis and its interpretation appropriate?

Yes

Are all the source data underlying the results available to ensure full reproducibility?

Yes

Is the study design appropriate and is the work technically sound?

Yes

Are the conclusions drawn adequately supported by the results?

Yes

Are sufficient details of methods and analysis provided to allow replication by others?

Yes

Reviewer Expertise:

Funtional food and nutraceuticals

I confirm that I have read this submission and believe that I have an appropriate level of expertise to confirm that it is of an acceptable scientific standard.

# Reviewer report: Reviewer response for version 1

**DOI:** 10.5256/f1000research.154999.r232442

**Published:** 2024-05-25

**Authors:** Dr Shivashankara AR

**Affiliations:** 1 Father Muller Medical College, Mangalore, India

This is a novel work on anticancer agents. The petroleum ether fraction of ethanolic leaf extract of *Annona reticulata* was found to have most potent anticancer effect. This study followed sound methodology and the conclusions drawn are adequately supported by the results. Data analysis is done properly. 

 Suggestions :

 1) Authors can include papers published in 2023 for citing

 2) Quality assurance method for the qualitative and quantitative analysis needs to be mentioned

Is the work clearly and accurately presented and does it cite the current literature?

Partly

If applicable, is the statistical analysis and its interpretation appropriate?

Yes

Are all the source data underlying the results available to ensure full reproducibility?

Yes

Is the study design appropriate and is the work technically sound?

Yes

Are the conclusions drawn adequately supported by the results?

Yes

Are sufficient details of methods and analysis provided to allow replication by others?

Yes

Reviewer Expertise:

Antioxidants and oxidants in health and disease; biochemical analysis of biological samples

I confirm that I have read this submission and believe that I have an appropriate level of expertise to confirm that it is of an acceptable scientific standard.

# Reviewer report: Reviewer response for version 1

**DOI:** 10.5256/f1000research.154999.r254403

**Published:** 2024-04-30

**Authors:** Veronique Seidel

**Affiliations:** 1 University of Strathclyde, Glasgow, Scotland, UK

This manuscript describes the phytochemical analysis and anticancer potential of the ethanolic extract of *Annona reticulata* (AR).

 Major comments:

- In the introduction, the authors should indicate if AR is used in traditional medicine.
- Several other references reporting on the anticancer activity of AR )and its phytoconstituents) should have been mentionned (eg: Ref-1; Ref-2; Ref-3; Ref-4; Ref-5.
- The authors should detail when the plant was collected (month/year) and include where a voucher specimen has been deposited (including its code number)
- GC-MS alone only putatively identifies compounds within a plant extract. A more reliable methodology would have been to use MS/MS.
- The anticancer potential of the ethanolic extract of AR leaves has previously been reported using HCT-116 cells. (Ref-6

Minor comments:

- References 1, 2 and 5 are too old and must be updated. The authors should quote GLOBOCAN 2022 for reference 1
- The preliminary phytochemical tests are standard tests. Please provide references for these testsThis manuscript describes the phytochemical analysis and anticancer potential of the ethanolic extract of *Annona reticulata* (AR).

Is the work clearly and accurately presented and does it cite the current literature?

Partly

If applicable, is the statistical analysis and its interpretation appropriate?

Yes

Are all the source data underlying the results available to ensure full reproducibility?

Yes

Is the study design appropriate and is the work technically sound?

Partly

Are the conclusions drawn adequately supported by the results?

Partly

Are sufficient details of methods and analysis provided to allow replication by others?

Partly

Reviewer Expertise:

Natural Products Research

I confirm that I have read this submission and believe that I have an appropriate level of expertise to state that I do not consider it to be of an acceptable scientific standard, for reasons outlined above.

## References

[1] : Induction of Mitochondria Mediated Apoptosis in Human Breast Cancer Cells (T-47D) by Annona reticulata L. Leaves Methanolic Extracts. *Nutr Cancer* .2016;68(2) : 10.1080/01635581.2016.1142583 305-11 10.1080/01635581.2016.1142583 26908199

[2] : Screening of promising chemotherapeutic candidates from plants against human adult T-cell leukemia/lymphoma (III). *J Nat Med* .2013;67(4) : 10.1007/s11418-013-0747-2 894-903 10.1007/s11418-013-0747-2 23397239

[3] : In vitro antiproliferativeactivity of Annona reticulata roots on human cancer cell lines. *Pharmacognosy Res* .2011;3(1) : 10.4103/0974-8490.79109 9-12 10.4103/0974-8490.79109 21731389 PMC3119276

[4] : Selective cytotoxicity of squamocin on T24 bladder cancer cells at the S-phase via a Bax-, Bad-, and caspase-3-related pathways. *Life Sci* .2006;78(8) : 10.1016/j.lfs.2005.05.068 869-74 10.1016/j.lfs.2005.05.068 16154156

[5] : Annonacin, a mono-tetrahydrofuran acetogenin, arrests cancer cells at the G1 phase and causes cytotoxicity in a Bax- and caspase-3-related pathway. *Life Sci* .2003;72(25) : 10.1016/s0024-3205(03)00190-5 2853-61 10.1016/s0024-3205(03)00190-5 12697268

[6] : Unveiling antioxidant and anti-cancer potentials of characterized Annona reticulata leaf extract in 1,2-dimethylhydrazine-induced colorectal cancer in Wistar rats. *J Ayurveda Integr Med* .2021;12(4) : 10.1016/j.jaim.2021.05.010 579-589 10.1016/j.jaim.2021.05.010 34674920 PMC8642649

# Response

**Published:** 2024-06-01

**Authors:** Vijetha Shenoy Belle

**Affiliations:** KASTURBA MEDICAL COLLEGE, MANIPAL, India

**Major comments:**

 1. In the introduction, the authors should indicate if AR is used in traditional medicine.

Response to reviewer’s comments:

 The ‘Introduction’ section of the manuscript has been modified as per the suggestions provided by the reviewer.

 2. Several other references reporting on the anticancer activity of AR) and its phytoconstituents) should have been mentioned (eg: Ref-1; Ref-2; Ref-3; Ref-4; Ref-5.

Response to reviewer’s comments:

 The references are cited in the introduction about *Annona reticulata.*

 3. The authors should detail when the plant was collected (month/year) and include where a voucher specimen has been deposited (including its code number)

Response to reviewer’s comments:

 The plant was collected in May 2020 and it was authenticated by Dr. Padma S, Department of Plant Science, Bangalore Central University, Bangaluru. The specimen voucher number was not provided by the authenticator. So we are attaching the  certificate provided by her.

*https://f1000research.s3.amazonaws.com/linked/653415.141542_Referee_Veronique_Seidel_v1.pdf*

 4. GC-MS alone only putatively identifies compounds within a plant extract. A more reliable methodology would have been to use MS/MS.

Response to reviewer’s comments:

  Due to constrain in the funds only GC-MS was done to identify the phytoconstituents in the extract.

 5. The anticancer potential of the ethanolic extract of AR leaves has previously been reported using HCT-116 cells. (Ref-6

Response to reviewer’s comments:

 This study (Ref 6) was conducted by the same corresponding author. Reference 6 was an *in-vivo *study. The current *in-vitro *study used ethanolic extract along with its fractions for its anticancer properties on HCT-116 cell lines along with details on bioinformatic analysis.

**Minor comments:**

 1.References 1, 2 and 5 are too old and must be updated. The authors should quote GLOBOCAN 2022 for reference 1. Change the references which are older.

Response to reviewer’s comments:

 New reference has been updated in the manuscript.

 2. The preliminary phytochemical tests are standard tests. Please provide references for these tests. This manuscript describes the phytochemical analysis and anticancer potential of the ethanolic extract of *Annona reticulata* (AR).

Response to reviewer’s comments:

 Reference 10, 11 are for all the preliminary phytochemical standard tests.
